# Highly Pathogenic Avian Influenza Virus A (H7N3) in Domestic Poultry, Saskatchewan, Canada, 2007

**DOI:** 10.3201/eid1509.080231

**Published:** 2009-09

**Authors:** Yohannes Berhane, Tamiko Hisanaga, Helen Kehler, James Neufeld, Lisa Manning, Connie Argue, Katherine Handel, Kathleen Hooper-McGrevy, Marilyn Jonas, John Robinson, Robert G. Webster, John Pasick

**Affiliations:** Canadian Food Inspection Agency, Winnipeg, Manitoba, Canada (Y. Berhane, T. Hisanaga, H. Kehler, J. Neufeld, L. Manning, C. Argue, K. Handel, K. Hooper-McGrevy, J. Pasick); Prairie Diagnostic Services, Saskatoon, Saskatchewan, Canada (M. Jonas); British Columbia Ministry of Agriculture and Lands, Abbotsford, British Columbia, Canada (J. Robinson); St. Jude Children’s Research Hospital, Memphis, Tennessee, USA (R.G. Webster)

**Keywords:** Influenza A virus, H7N3 subtype, highly pathogenic, viruses, influenza, poultry, wild birds, Canada, dispatch

## Abstract

Epidemiologic, serologic, and molecular phylogenetic methods were used to investigate an outbreak of highly pathogenic avian influenza on a broiler breeding farm in Saskatchewan, Canada. Results, coupled with data from influenza A virus surveillance of migratory waterfowl in Canada, implicated wild birds as the most probable source of the low pathogenicity precursor virus.

Wild aquatic birds of the orders Anseriformes and Charadriiformes are the natural reservoir for influenza A viruses (*1*) and are thought to serve as a source of virus that leads to outbreaks in domestic poultry. However, direct evidence for this suggestion is often difficult to demonstrate.

On September 22, 2007, a broiler hatching egg operation near Regina Beach, Saskatchewan, Canada, experienced a sudden increase in deaths (140 [36%] of 390 birds) in a barn that housed 24-week-old roosters. The premises contained 53,000 birds of multiple ages housed in 10 confinement barns. On September 23, deaths in the rooster barn increased to 240 (62%). Postmortem examination findings, which showed lesions compatible with highly pathogenic avian influenza (HPAI), resulted in a Canadian Food Inspection Agency team being dispatched to the premises. The farm was placed under quarantine, and specimens were submitted to the regional Avian Influenza Network laboratory in Saskatoon, Saskatchewan, and the National Centre for Foreign Animal Disease in Winnipeg, Manitoba, for diagnosis. A complete account of the index premises, disease control actions, and description of the Saskatchewan poultry industry is online at www.inspection.gc.ca/english/anima/heasan/disemala/avflu/2007sask/repsaske.shtml.

## The Study

Six pools (5 samples per pool) of cloacal swab specimens, 6 pools (5 samples per pool) of oropharyngeal swab specimens, 6 pools of 10% (wt/vol) tissue (heart, liver, lung, and spleen), 6 pools of intestine homogenates, and 2 pools of brain homogenates were tested by using real-time reverse transcription–PCR (RT-PCR) assays specific for the influenza A virus matrix gene (*2*), H5 and H7 hemagglutinin (HA) subtype genes (*2*), and avian paramyxovirus serotype-1 matrix gene (*3*). Virus isolation was performed by using embryonating chicken eggs according to international standards (*4*). RT-PCR of swab and tissue samples showed positive results for influenza A matrix but negative results for H5 and H7 subtypes and avian paramyxovirus serotype-1.

Because of apparent inconsistencies between these initial results and clinical signs observed on the farm, further analyses were conducted by using conventional RT-PCR assays with universal primers designed to amplify the complete HA gene and the 9 neuraminidase gene subtypes of avian influenza virus (*5*). Results from these ancillary tests showed evidence for an avian influenza virus (H7N3), which was subsequently confirmed by virus isolation and subtyping by hemagglutination-inhibition and neuraminidase-inhibition assays (*4*).

Viral isolates grew well in the chicken host, producing HA titers as high as 1,024. The derived amino acid sequence of the HA_0_ cleavage site, PENPKTTKPRPRR/GLF, (underlined amino acids indicate a 6-aa insert) conformed to the definition of the World Organisation for Animal Health for HPAI virus (*4*). Intravenous inoculation of 4- to 6-week-old chickens (*4*) with isolate A/chicken/Saskatchewan/HR-00011/2007 resulted in all birds dying within 24 hours, giving an intravenous pathogenicity index of 3.0. This finding confirmed the molecular pathotype. Tissues from dead roosters showed specific influenza A virus immunolabeling in all organs examined, including the central nervous system, a characteristic of HPAI.

Negative real-time RT-PCR results for H7 were explained by the presence of 8-nt substitutions within the primer and probe target sites: 2 and 1 in the forward and reverse primers, respectively, and 5 in the probe. This real-time RT-PCR assay for H7 (*2*) did not detect several H7 viruses subsequently isolated from wild birds in 2007, a finding that has also been reported by Xing et al. (*6*).

Epidemiologic investigations conducted on poultry farms surrounding the index premises, including 6 farms located within the 3-km surveillance zone, showed no evidence of avian influenza virus infection. Sharing of equipment, movement of employees among the barns, and lack of designated footwear or clothing for each employee on the index farm increased the likelihood of inadvertent introduction of environmental contaminants. The barns used a municipal water source, but during high demand periods surface water from a dugout located ≈380 m from the breeder barns was also used. This water was routinely filtered and treated with ozone, but a failure of the ozonater was reported during July. Several small natural water bodies are also located near the premises, the closest being 1,100 m away. Last Mountain Lake, which has a length of 80 km and a waterfowl staging area at its northern end, is located 5.5 km away.

Serologic testing was conducted to evaluate the length of time an avian influenza virus (H7N3) had been circulating on the premises. Of serum samples obtained from 24-week-old roosters on September 23, 62% (18/29) had antibodies against avian influenza virus nucleoprotein (NP) (*7*); all were negative for antibodies against H7 (*4*). Analysis of serum samples from surviving roosters 3 days later showed that 90% (18/20) had antibodies against NP and 84% (16/19) had antibodies against H7. In contrast, 100% (21/21) of 32-week-old breeder hens had antibodies against NP and 93% (14/15) had antibodies against H7, and 95% (19/20) of 55-week-old breeder hens had antibodies against NP and 87% (14/16) had antibodies against H7. These results suggest that breeders had been infected longer than roosters.

**Figure 1:**
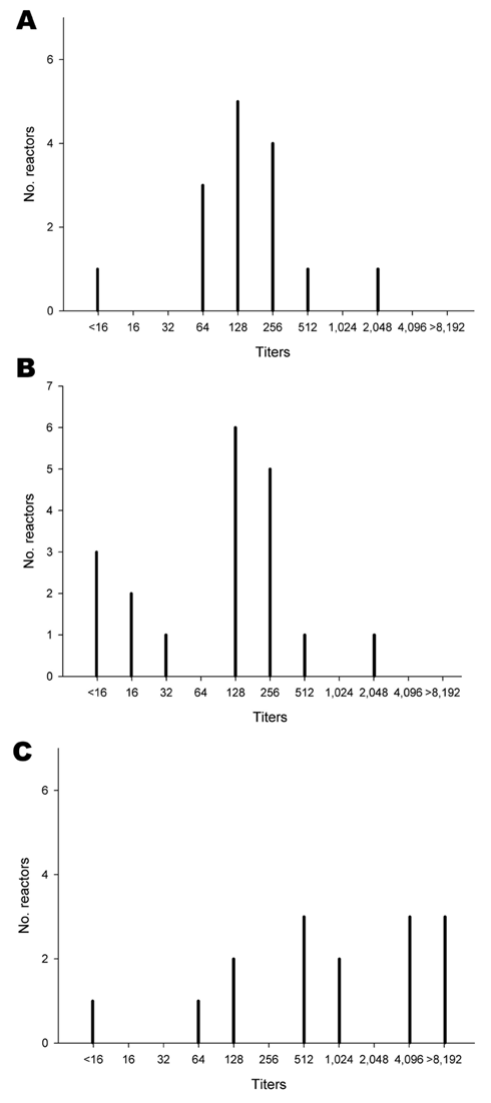
Avian influenza virus H7-specific antibody titers of serum samples from 55-week-old breeder chickens (A), 24-week-old roosters (B), and 32-week-old breeder chickens (C), Saskatchewan, Canada, September 26, 2007. Titers of individual birds were determined by the ability of 2-fold serial serum dilutions to inhibit agglutination 0.5% (vol/vol) chicken erythrocyte suspensions by 4 hemagglutination inhibition units of avian influenza virus (H7N3) A/chicken/British Columbia/2004.

Serum samples that had been obtained from the 55-week-old flock on May 11, 2007, and the 32-week-old flock on June 8, 2007, were negative for antibodies against NP, which indicated that virus introduction occurred subsequently. Although samples from 55-week-old breeders had high H7 antibody titers, none of these birds showed overt clinical signs, which implied exposure to an avian influenza virus (H7N3) with low pathogenicity. Of note, ≈25% of 32-week-old breeders were clinically ill on September 28 (5 days after the initiation of quarantine), despite having some of the highest H7-specific antibody titers (Figure 1).

**Figure 2:**
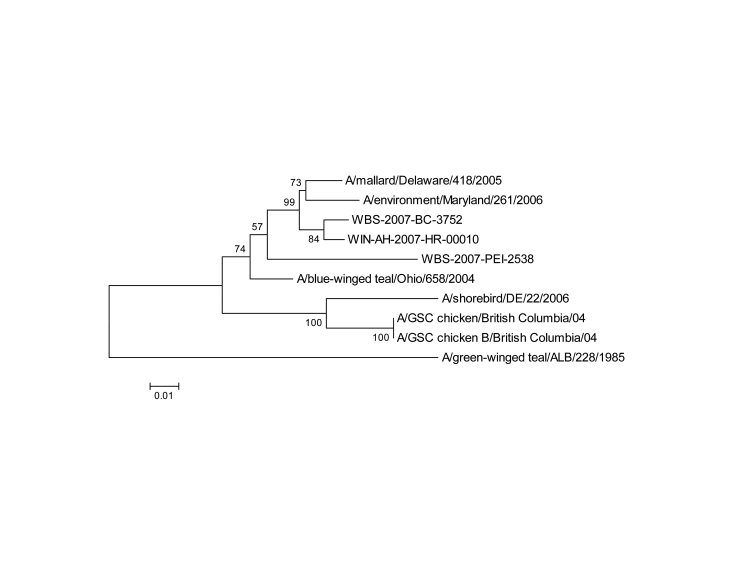
Phyogenetic analysis of avian influenza virus H7 (A) and N3 (B) genes. Trees were generated with MEGA software (*8*) by using the neighbor-joining method (*9*). Evolutionary distances were computed by using the method of Nei and Gojobori (*10*). Percentage of replicate trees in which the associated taxa clustered together in the bootstrap test (1,000 replicates) is shown next to the branches. Scale bars indicate substitutions per site.

Phylogenetic analysis (Figure 2; Tables 1, 2) showed a close relationship of Saskatchewan/2007 H7N3 with recent North American H7 subtype viruses of free-flying waterfowl origin (*11*). Several of these viruses were isolated during an avian influenza surveillance program that had been coordinated since 2005 by the Canadian Cooperative Wildlife Health Centre. Although the wild bird surveillance program in Canada did not detect H7 viruses in 2005 (*12*) or 2006, a conclusion based on characterization of viruses that were isolated from all real-time RT-PCR swab samples positive for virus matrix gene, several H7 virus isolates were obtained in 2007. Most of these viruses were isolated from birds sampled in the neighboring provinces of British Columbia, Alberta, and Manitoba. The HA gene of Saskatchewan/2007 clusters with these 2007 wild bird isolates but not with the HA gene of A/chicken/British Columbia/2004 (H7N3), which was responsible for the HPAI outbreak in British Columbia. This finding further supports the hypothesis that the Saskatchewan/2007 isolate was of wild bird origin.

**Table 1:** Comparison of 8 gene segments of avian influenza virus (H7N3) A/chicken/Saskatchewan/HR-00011/2007 with influenza virus genes from GenBank with highest sequence identity, Saskatchewan, Canada, 2007*

Gene	Nucleotide identity	%	Amino acid identity	%
PB2	A/laughing gull/Delaware/42/2006 (H7N3)	98	A/mallard/Ohio/656/2002 (mixed)	98
PB1	A/mallard/Alberta/149/2002 (H2N4)	98	A/mallard/Alberta/149/2002 (H2N4)	99
PA	A/snow goose/Maryland/353/2005 (H6N1)	98	A/mallard/Ohio/249/98 (H6N1)	99
H7	A/mallard/Delaware/418/2005 (H7N3)	96	A/environment/Maryland/566/2006 (H7N9)	97
NP	A/environment/Maryland/1176/2005 (H3N6)	98	A/GSC_chicken/British Columbia/2004 (H7N3)	99
N3	A/mallard/Delaware/418/2005 (H7N3)	98	A/environment/Maryland/261/2006 (H7N3)	98
M1	A/mallard/Maryland/1131/2005 (H12N5)	98	A/chicken/Korea/S6/2003 (H3N2)	98
M2	A/chicken/Singapore/93 (H10N5)	98	A/mallard duck/Alberta/376/85 (H2N3)	98
NS1	A/mallard/Alberta/30/2001 (H4N8)	98	A/pintail/Alaska/779/2005 (H3N8)	98
NS2	A/blue-winged teal/Louisanna/240B/88 (H4N6)	99	A/mallard/Delaware/418/2005 (H7N3)	98

*PB2, RNA polymerase subunit B2; PB1, RNA polymerase subunit B1; PA, RNA polymerase subunit A; H7, hemagglutinin 7; NP, nucleoprotein; N3, neuraminidase 3; M1, matrix protein 1; M2, matrix protein 2; NS1, nonstructural protein 1; NS2, nonstructural protein 2.

**Table 2:** Comparison of 8 gene segments of avian influenza virus (H7N3) A/chicken/Saskatchewan/HR-00011/2007 with 2 recent viruses (H7N3) isolated from wild waterfowl, Saskatchewan, Canada, 2007*

Gene	A/American black duck/New Brunswick/2538/2007			A/Canada goose/British Columbia/3752/2007	
	% nt identitiy	% aa identity		% nt identity	% aa identity
PB2	92	97.8		92	97.8
PB1	97	99.2		97	99.1
PA	98	95.5		97	95.4
H7	96	96.3		97	97
NP	94	97.4		94	97.2
N3	96	98.3		98	98.3
M1	96	98.8		97	98.8
M2	98	97.9		99	100
NS1	80	68.7		80	69.1
NS2	80	82.5		80	82.5

*GenBank accession nos. EU500841–EU500864. PB2, RNA polymerase subunit B2; PB1, RNA polymerase subunit B1; PA, RNA polymerase subunit A; H7, hemagglutinin 7; NP, nucleoprotein; N3, neuraminidase 3; M1, matrix protein 1; M2, matrix protein 2; NS1, nonstructural protein 1; NS2, nonstructural protein 2.

## Conclusions

Potential breaches in biosecurity and proximity of the farm to a waterfowl habitat point to wild aquatic birds as the most likely virus source. Serologic evidence suggests that a low pathogenicity avian influenza virus (H7N3) circulated among breeder hens before roosters were exposed. Infection of 24-week-old roosters was likely associated with their movement into breeder barns on September 13, 18, and 19. This finding coincided with evolution of an HPAI virus by a process that may have involved nonhomologous recombination similar to that described for the HPAI (H7N3) outbreak in British Columbia (*13*). The origin of the 6-aa insert within the HA_0_ cleavage site remains speculative; a hypothetical protein of *Gallus gallus* (GenBank accession no. XM_424122) was 1 notable potential donor. The findings of this and other studies (*6*) emphasize the need for continually monitoring HA subtype-specific real-time RT-PCR assay performance, particularly when used in national avian influenza surveillance programs.
